# Barriers for why pregnant women do not visit a dentist on a regular basis: using group concept mapping methodology

**DOI:** 10.1080/00016357.2023.2283198

**Published:** 2024-03-26

**Authors:** Karoline Winckler, Marianne Uggen Rasmussen, Jeannet Laugenborg, Kathrine Hansen Bukkehave, Heidi Fischer, Berit Lilienthal Heitmann, Eva Ejlersen Wæhrens

**Affiliations:** a The Parker Institute, Bispebjerg and Frederiksberg Hospital, University of Copenhagen, Copenhagen, Denmark; b Department of Pediatrics, Gynaecology and Obstetrics, Nykøbing Falster Hospital, Region Zealand, Denmark; c Department of Obstetrics and Gynecology, Copenhagen University Hospital Herlev, Herlev, Denmark; d Department of Obstetrics and Gynaecology, Holbæk Hospital, Region Zealand, Denmark; e Department of Public Health, Section for General Practice, University of Copenhagen, Copenhagen, Denmark; f Occupational Science, User Perspectives and Community-based Research, Department of Public Health, University of Southern Denmark, Odense, Denmark

**Keywords:** Dental care experience, pregnancy, prenatal care

## Abstract

**Objectives:**

Periodontitis in pregnancy represents a significant, but often overlooked challenge due to its association to adverse pregnancy (preeclampsia and gestational diabetes) and birth related outcomes (preterm birth and low birth weight). The overall study aim was to identify, organize, and prioritize barriers influencing dental visits among Danish pregnant women not seeing a dentist on a regularly basis.

**Materials and methods:**

Participants were pregnant women screened at weeks 11–13 of gestation, and were recruited if they were not seeing a dentist regularly. The study was conducted at Holbæk and Nykøbing Falster Hospital in Region Zealand, Denmark. The Group Concept Mapping (GCM) approach was applied. The pregnant women participated in brainstorming (*n*=18), sorting (*n*=20), and rating (*n*=17) the seating question ‘*Thinking as broadly as you can, please list all barriers of importance to you for not seeing a dentist on a regular basis*’.

**Results:**

A total of 38 unique barriers were identified, organized, and prioritized online. The multidimensional scaling analysis involved 10 iterations and revealed a low stress value of 0.21. A cluster solution with five clusters including ‘economic reasons’, ‘lack of priority’, ‘lack of time and energy’, ‘no problems with teeth’, and ‘dental fear’, was discussed and interpreted at a validation meeting.

**Conclusions:**

Five overall clusters explaining barriers for not seeing a dentist regularly were revealed. Of the five clusters, ‘economic reasons’ and ‘lack of priority’ were rated as the most important clusters. Accordingly, such barriers should be considered in the planning of future strategies of dental care during pregnancy.

## Introduction

Pregnancy is a condition with changed hormonal balance increasing the risk of gingivitis and periodontitis [1]. Periodontitis during pregnancy is a significant public health issue, with an estimated prevalence of 40% [2], due to its association with chronic diseases, adverse pregnancy, and birth related outcomes, including preterm birth [3–5]. Salivary microbiota have been found associated with gingivitis, the initial stage of periodontitis, and development of gestational diabetes [6]. The accompanying inflammatory condition in the tissues surrounding the teeth, caused by periodontitis may lead to a state of low-grade inflammation, which causes metabolic disturbances that may increase the risk of complications during pregnancy [4,7,8]. Optimal dental care constituting both professional tooth cleaning (elimination of calculus and/or subgingival plaque) and a good daily oral hygiene routine, is important, especially during pregnancy [9,10]. International recommendations for pregnant women are available, which provides guidance on how to ensure oral health [10]. Pregnancy is considered an ideal time-window to establish and present educational and preventive initiatives, as pregnant women are generally more receptive to information about the health of their babies’ and their own well-being, and to implementing better health practices [11].

In Denmark, all dental visits are paid through the Danish Healthcare system until the age of 18 years. After the age of 18 years, the Danish Healthcare system provides a subsidiary part of around 20% on patient’s dental health care expenses. Information on oral health habits including dental visits among pregnant women is not available in Denmark, but national data from the Danish Dentist Association indicate that up to 40% of all women in childbearing age do not attend a dentist on a regular basis although recommendations are available [12]. Previous studies, from Portugal, Spain, France, the United States, and Malaysia, have analyzed some of the barriers and facilitators that influence dental care during pregnancy [13–15]. Themes, that have been identified as relevant barriers for not seeing a dentist on a regular basis, include psychological and physical conditions, such as discomfort associated with dental treatment, either due to the treatment itself or the position in the dental chair of the pregnant woman, low attributed importance of oral health, fear of/anxiety towards dental treatment, financial barriers, and time constraints. Identifying the most relevant barriers for not seeing a dentist on a regular basis among pregnant women are of critical importance to improve intervention strategies for oral health during pregnancy, which will have a positive impact on pregnancy outcome. The findings of this study are therefore important for the design of potential intervention strategies, including for the implementation of the findings and recommendations of the PROBE study, which the present study is part of, and which investigates the effects of periodontal treatment on pregnancy and birth related outcomes.

To better understand why pregnant women may not attend regular dental visit, the aim of this study was to identify, organize, and prioritize barriers influencing dental visits among Danish pregnant women not seeing a dentist regularly, thereby hopefully providing more attention to this area, implement new guidelines and ultimately improving oral health during pregnancy.

## Materials and methods

### Participants

Participants were to fulfill the following inclusion criteria: Pregnant women attending their routine nuchal translucency scan at week 11–13 of gestation at either Department of Obstetrics and Gynaecology at Holbæk or Nykøbing Falster Hospital in Region Zealand, Denmark reporting not seeing a dentist on a regularly basis. The criterion of not seeing a dentist on a regular basis was assessed in a structured questionnaire with the following two questions being posed: (1) how many times have you seen the dentist within the past 5 years (number of times), and (2) when did you last see a dentist (≤6 months, >6 months to 1 year ago, >1 to 1½ years ago, >1½ to 2 years ago, >2 to 3 years ago or >3 years ago). Women reporting that they visited a dentist <2 times during the past 5 years or that their last visit happened >1½ year ago, met the inclusion criterion.

Research staff screened 63 potential participants during April and May 2021. Eligible women were informed about the study including the need to participate in at least one of three elements: brainstorming, sorting and rating, and validation of data. The women received an electric tooth brush for their participation.

### Patient and public involvement

In the present study, there was no patient and public involvement in the design, reporting, and dissemination of the study. In accordance with the Group Concept Mapping method applied, the participants were involved in parts of the data analysis.

### Study design

The study was based on Group Concept Mapping (GCM), a methodology for generating and structuring ideas on a specific topic, involving a type of integrative mixed method participatory approach, combining qualitative and quantitative approaches to data collection and analysis [16–18]. Participants in GCM studies are involved in several steps of the research process, including generating ideas, structuring statements, and/or interpreting the map. The GCM process may involve face-to-face group sessions, online participation, or both [16,19,20]. The final results are illustrated in maps, where ideas on the specific topic are organized thematically.

In the present study the following phases were included in the structured GCM process: (1) preparing for GCM, (2) generating the ideas (brainstorming), (3) structuring the statements (sorting, labelling, and rating), (4) GCM analysis (data analysis), and (5) interpreting the map (validation) [19]. These five phases provided a structure for the process. Brainstorming, sorting, labelling, rating, and generation of cluster rating map were performed using the Concept System (CS)^®^ Groupwisdom™ software, designed to support each step in the GCM process (CS Incorporated, 2019).

### Study procedures

#### Participant demographics

Eligible pregnant women accepting the invitation to participate provided information regarding age, marital status, number of children and pregnancies, education, employment, and information about their partners’ age, education, and employment through an online questionnaire conducted in the research electronic data capture (REDCap) program (Table 1).

**Table 1:** Demographic data of the participants (*n* = 23).

Age, years (median, range)	29 (20–42)
Partners age (years, range)	33 (23–44)
Civil status (%)	
Single	17
Cohabiting	83
Number of previous pregnancies (%)	
0	30
1	17
2	52
Number of children (%)	
0	48
1	39
2	13
Participant’s education (%)/partner’s education (%)	
Primary school	17/13
Vocational education	4/26
Highschool	26/9
Short higher education (<3 years)	13/9
Medium higher education (3–5 years)	30/17
Long higher education (>5 years)	9/17
Unknown partner education	9
Participant’s employment (%)/partner’s employment (%)	
Full-time employed	35/70
Part-time employed	9/0
Part-time employed (flex performance)	9/0
Student	17/22
Cash benefit recipient	13/0
Unemployed	9/9
Stay-at-home (no income)	4/0
Other	4/0

#### Preparing for GCM

Before initiating the data collection, a focus prompt was formulated and piloted. The final version was: *‘Thinking as broadly as you can, please list all barriers of importance to you for not seeing a dentist on a regular basis’*.

#### Generation of ideas (brainstorming)

Participants received an e-mail with a link to individual online participation using Groupwisdom software. In the software, the participants were instructed to brainstorm with as many brief continuations as possible to the focus prompt. They were reminded to keep each sentence/idea short containing only one meaning (for instance ‘dental visits are expensive’). Based on the participants’ input an overall list of ideas was generated. For further information, see the section on data analysis.

#### Structuring the statements (sorting and rating)

Again, the participants received an e-mail containing information about the sorting and rating tasks as well as a new link to Groupwisdom. The first task was to sort all the ideas generated during the brainstorm into piles and to label each pile. This was an individual task performed according to individual preferences. Next, each participant rated the importance of each idea on a four-point ordinal scale; a score of one being ‘*Unimportant*’ and a score of four being ‘*Very important*’. Finally, each participant rated the need for a change related to each idea on a two-point scale; ‘*yes*’ and ‘*no*’.

#### GCM analysis (data analysis)

Based on the sorting and rating, a Cluster Rating Map was generated using the Groupwisdom software to be presented at the face-to-face validation meeting in the author group in phase five. For further information, see the section on data analysis.

#### Interpreting the map (validating)

The validation meeting was conducted in three steps in which the participants were individually asked to consider: (1) whether each of the statements were placed in a cluster that best matched the meaning of the other statements in the cluster, (2) the number of clusters, and (3) if the cluster labels illustrated the theme of the cluster. All suggestions were discussed and consensus reached regarding cluster names, content, and numbers of clusters.

## Data analyses

### Data from REDCap

Demographic data on all participants taking part in the brainstorming phase were collected through REDCap. These data were analyzed using IBM SPSS statistics, and values were presented as median (minimum–maximum) for non-normally distributed continuous variables, and frequencies (proportions) for categorical variables (see Table 1).

### Data from group concept mapping

Groupwisdom software was used to perform data analyses based on the ideas derived from the brainstorming. The analyses were conducted in several steps. Ideas gathered were consolidated; identical (i.e. redundant) ideas were manually identified by the first and second authors and removed after consensus was reached, and, if needed, ideas were revised to clarify the meaning. The remaining ideas were kept in Groupwisdom in preparation for phases three and four.

Individual participant data from phase three were to be included in the cluster analysis if more than 75% of the ideas were sorted and if <5 ideas remained unrated [21]. Based on the sorting and rating of ideas, multidimensional scaling analysis and cluster analysis were performed by the first, second, and last authors in which related ideas were grouped into clusters [18]. During this process, several cluster solutions were generated and the one that matched the data the best (i.e. the cluster solution representing sufficient details on the topic) was applied, creating the Cluster Rating Map (phase four) [18]. Within the multidimensional scaling analysis, the stress value was used to indicate ‘goodness of fit’. A stress value of <0.39 is considered to indicate congruence between the raw data and processed data [18]. Based on the labels provided by the participants in phase three, cluster labels were suggested by Groupwisdom. Besides illustrating the labelled clusters in relation to each other, the importance of ideas included in each cluster was also depicted by the number of layers in each cluster, based on median values for importance ratings given for each idea in the cluster.

## Results

Through GCM, participants identified, organized, and priori-tized barriers for not seeing a dentist on a regular basis. Statements were generated and represented a complex and broad perspective on the topic. Multidimensional scaling analysis of the participants’ sortings and ratings resulted in a cluster rating map which provided the basis for the overall conclusion. In the following sections, the demographic data of the participants and the data derived from the GCM process, is presented.

### Participants

Based on screening, 30 pregnant women not seeking dental care on a regular basis were identified as potential participants. Of these, 23 pregnant women accepted the invitation and were involved in at least one phase of the GCM process. In total, 18 (78%) were involved in the brainstorming phase, 20 (87%) in sorting and 17 (74%) in rating the importance of each statement, and 10 (43%) in rating each statement in terms of the need for a change. These women also responded to the online questionnaire about demographic information (Table 1).

### Group concept mapping data

A total of 30 statements were initially generated during the brain storming phase. Several of these (*n* = 20), included more than one meaning and were therefore split into two or three separate statements. There were also redundant ideas (*n* = 10), and after removing these, 38 unique statements remained and were included in the sorting and rating phase.

Data from the sorting phase were excluded for four participants, as they sorted too few statements or sorted all the statements in one pile. Data for the sorting phase was based on the remaining 17 participants. These participants sorted the statements into between 3 and 10 piles (median = 5).

**Figure 1:**
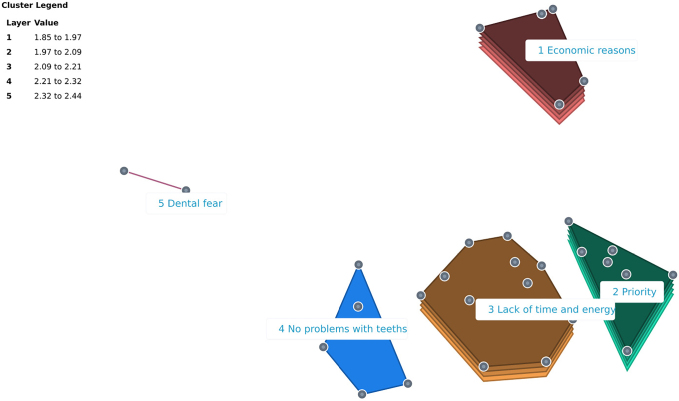
Cluster rating map with five clusters. Legend: Each point on the map represents a statement. Proximity of statements on the map indicates how related they are. Statements positioned closer to each other were more often sorted together, indicating that they concern aspects of the same topic. The size and shape of each cluster are formed by the position of the statements. The height of a cluster signifies its relative importance, with higher clusters (i.e. the number of layers) containing statements being rated as more important.

The multidimensional scaling analysis involved 10 iterations and revealed a low stress value of 0.21. In the cluster analysis, solutions with three to seven clusters were thoroughly examined to determine the cluster solution representing sufficient details on the topic. The cluster solution with five clusters was preliminary chosen to be further discussed at the validation meeting. The five clusters, each containing between two and 14 statements, are presented in a cluster rating map (Figure 1).

An online validation meeting was scheduled and a subset of representative participants was invited to interpret and validate the results. Unfortunately, only one of the invitated participants was able to participate in the validation meeting. The validation meeting was therefore held within the author group, where five of the authors participated.

At the face-to-face validation meeting, discussions led to consensus about the location of the statements, and 9 (23.7%) statements were moved between clusters. The number of clusters were maintained and each cluster in the revised map now contained between 2 and 15 statements (Table 2 and Supplementary Appendix A). Furthermore, the author group suggested changes to the cluster labels, based on the content of each cluster (Table 2). The content of each cluster is summarized in Table 3.

**Table 2:** Key Concept cluster statistics.

Key concept clusters within the conceptual model	Number of Statements *n* (%)	Rating of importance	Rating of need for change
		Cluster median (range)	Statements with high ratings of importance and need for change* *n* (%)
1. Economic reasons	10 (26.3)	2.5 (1–3)	3 (30.0)
2. Lack of priority	15 (39.5)	2 (1–3)	2 (13.3)
3. Lack of time and energy	8 (21.0)	1.5 (1–3)	1 (12.5)
4. No problems with teeth	3 (7.9)	1.5 (1–2)	0
5. Dental fear	2 (5.3)	1 (1)	0

*Note*. The cluster median is calculated based on median values of ratings of importance on a four-point scale for each statement within each cluster. Range statistics represent the lowest and highest median values, respectively, for ideas within a cluster. The proportion of participants that have responded a need for change is presented in %.

* Statements with a median rating of importance ≥3 combined with a need for changes defined as ≥70% of the participants rating ‘yes’.

**Table 3:** Brief description of the five clusters.

Cluster	Summary of content
Economic reasons	Participants stated that they find dental care expensive and although they would like to receive dental care, they found themselves unable to afford it, as they considered themselves financially challenged. The uncertainty of not knowing the exact cost of the dental treatments they might need was also a concern for not seeking dental care.
Lack of priority	Participants stated that they have never gotten ‘into the routine’ of prioritizing dental check-ups and were unable to fit it into their schedule. Instead, they would postpone or forget to make a dental appointment. Although they were aware that it is important, they considered it as something that they didn’t have to do.
Lack of time and energy	Participants stated that they were lacking the time, energy, and motivation to seek dental care while balancing a busy everyday life with work and children.
No problems with teeth	Participants stated that they do not seek dental care, since they have no problems with their teeth.
Dental fear	Participants stated that they do not seek dental care because of dental fear and previous bad experiences with a dentist.

Of the five clusters, ‘economic reasons’ (median = 2.5) and ‘lack of priority’ (median = 2) were rated as most important (Table 2). ‘Dental fear’ was rated of least important (median = 1). Six statements displayed a high rating of importance (median ≥3) combined with a need for changes (≥70% rating ‘yes’), distributed within three clusters. Hence, in cluster 1 concerning ‘economic reasons’, the participants considered three statements important and in need of a change (‘primarily because it is expensive’, ‘I don’t think I can afford it’, and ‘You never know how much money it would end up being’). Similarly, in cluster 2 concerning ‘lack of priority’, they rated the statements ‘I just don’t get it done’ and ‘I forget to make the time for it’ as highly important to address. Finally, in cluster 3 concerning ‘lack of time and energy’, the statement ‘I forget my self’ was considered important and something to be addressed.

## Discussion

### Main findings

The present study investigated and revealed barriers for not seeing a dentist on a regular basis among pregnant Danish women. Based on the GCM approach, five clusters emerged covering 38 unique statements representing reasons for not seeing a dentist: ‘economic reasons’, ‘lack of priority’, ‘lack of time and energy’, ‘no problems with teeth’, and ‘dental fear’. Of these, economic reasons and problems related to giving priority to dental visits were rated as the most important and dental fear as the least important reasons for not seeing a dentist. The participants were also asked to rate statements according to the need for change to address the reasons for not seeing a dentist. Economic reasons and insufficient prior-itization of dental visits were, together with dental fear, rated as the main barriers that needed to be addressed. The rating of dental fear was, however, only based on two statements as explained above.

### The five clusters

The five clusters represent the concretization of the phenomenon explored in this study; that many pregnant women are not seeking dental care, although it is widely acknowledged, that optimal dental care is especially important during pregnancy [5]. The clusters and statements within each cluster provide a deeper understanding with regards to what determined the women’s choice of not seeking dental care regularly. The clusters illustrated the complexity of the barriers related to the women’s decisions. Especially for the clusters ‘lack of priority’ and ‘lack of time and energy’ this complexity was clear. The interpretation of the generated statements within these clusters was particularly difficult. This was also confirmed at the validation meeting, held within the author group. There seems to be a clear distinction between not prioritizing regular dental visits because other things are considered more important and just not having the time and energy to seek dental treatment regularly. The cluster ‘lack of time and energy’ seems therefore to include social and psychological aspects. Knowledge gained from the cluster analyses is valuable in relation to understanding what measures are needed to ensure that more pregnant women will choose to seek dental care in the future.

### Comparison to other studies

Our study is the first study to identify, organize, and prioritize barriers influencing dental visits among Danish pregnant women not seeing a dentist on a regularly basis from two low socioeconomic status areas in Denmark. In addition, the participants can be considered as vulnerable, since the pregnancy in itself is considered a vulnerable condition, and also as the participants have not seen a dentist on a regular basis.

The findings of this study are relevant and important to better understand the complexity of some of the barriers and facilitators for why women in the child bearing are not seeing a dentist regularly, especially in Region Zealand. As mentioned, the present study is part of the PROBE study, a controlled intervention study, which is currently collecting data from pregnant women with periodontitis in Region Zealand with the aim of investigating the effect of periodontal treatment during pregnancy on adverse pregnancy and birth related outcomes. The findings of the current study is therefore crucial in terms of gaining insight into dental habits to better implement the findings afterwards. The study’s methodological approach using Group Concept Mapping, a mixed methods approach that engage the participants throughout the process, provides a more nuanced approach and therefore a better understanding of the complexity of the various barriers for not seeing a dentist, including the five clusters and 38 expressed statements by the participants. This understanding will be useful when the results and conclusions of the PROBE study are to be implemented.

Hence, results from a recent systematic review suggested a lack of knowledge of determinants for the use of dental services by pregnant women [22]. The authors concluded that many questions remain unanswered in particular with regard to psychosocial issues including beliefs and values. Another systematic review was therefore proposed to study in more detail factors already identified in qualitative studies [13]. That review found 14 themes related to barriers and facilitators to dental care during pregnancy that interacted in a complex matter: physiological conditions, low importance put on oral health, negative stigma regarding dentistry, fear of dental treatment, mobility and safety, financial barriers, employment status, time constraints, lack of social support, lack of information, health professionals’ barriers (the professionals were not comfortable with treating the pregnant women or they advised them to return after the baby’s birth), family and friends’ advice on not seeing a dentist during pregnancy because of medical restrictions, and beliefs and myths about the safety of dental treatment. The latter theme seemed to be the most important and a significant barrier for oral health and treatment during pregnancy, not only for the pregnant women but also for the dentist and for health professionals. Highlighting and finding the most relevant barriers for not seeing a dentist on a regular basis is of outmost relevance to overcome. Our findings show that economy and priorities are key barriers for not seeing a dentist regularly, which corresponds with previous studies [13].

One previous study found dental fear and lack of information about oral health during pregnancy as the dominant barriers for inadequate oral health and dental service during pregnancy [23]. Two reviews have previously investigated whether dentists, general practitioners, midwives, and obstetricians/gynecologists have any doubts or fears about dental care during pregnancy [24,25]. They found a general concern about dental care during pregnancy, especially with regard to the use of X-rays and prescriptions. Our study found dental fear as a relevant barrier although not the most important, and did not investigate barriers from the dentist’s perspective.

Changing priorities are difficult but it seems that knowledge regarding pregnant women’s oral health and the developmental effects of the baby are primary facilitators for the use of services, which has been concluded in similar studies [13]. Quantitative studies have found that pregnant women who recognize the connection between oral health and pregnancy use dental services more often [26,27]. The two primary barriers for not seeing a dentist on a regular basis found in the present study might reflect the socioeconomic status of the pregnant women. Lack of priorities and economy could be indicators of a subgroup of patients with less mental and physical resources. Our results correspond well with other studies and highlights the need for more focus on the area and maybe even including dental care and guidelines on optimal daily oral health in the antenatal care program. However, whether attending a dentist on a regular basis or an optimal daily oral health routine is the most important method of good oral health, or a combination of both, is a still an ongoing debate.

Future studies could address the promoting factors for why people do see a dentist on a regular basis. That would give interesting information and knowledge on what could be emphasized within the system.

### Strengths and limitations

In this study, it was considered a strength to employ GCM methodology and a suitable online tool to obtain knowledge from a subgroup of participants. In general, some of the benefits of GCM is the generation of data with high validity, reliability, and completion percentages [28]. GCM represents a mixed methods approach with the integration of qualitative and quantitative data [20] to an extent that blur the distinction between qualitative methodologies [18]. The unique feature of the GCM approach values the voice and engagement of participants, who are essential in the generation of data, the data analyses, and the validation of the results. Finally, the GCM process and results are visualized in maps, which facilitates the communication and dissemination of results to a broader audience.

With the aim of generating knowledge from a group of pregnant women not seeking dental care during pregnancy, some women could potentially feel uncomfortable or stigma-tized to share factors and barriers for not seeing a dentist on a regular basis, in a public setup. By employing GCM the brainstorming, sorting, and rating phases could be conducted online and anonymized.

The potential participants in the study being pregnant and not seeing a dentist on a regular basis, could be considered vulnerable. This was mirrored in a low response rate and in the willingness to contribute. Moreover, several reminders to the participants were required. The included women may therefore be considered a difficult group to target, which Table 1 also indicates in terms of the socioeconomic status, including educational level and employment. Another limitation, which is closely related, is the fact that it was not possible to gather a sufficient number of participants for the validation meeting and that the validation meeting accordingly was held within the author group. This may have influenced the results and conclusions at the validation meeting. However, performing a validation meeting is not a mandatory part of the GCM methodology, but may be added to the process [18]. Thirdly, it cannot be excluded that some participants would have dental visits later in their pregnancy. However, as shown above, a relative limited percentage visit the dentist on a regular basis, and there is no reason to believe that accommodating these potential later visits would change the main results of this study.

The study showed that the participants can have many reasons for not seeing a dentist, on a regular basis. Obviously, care should be taken when attempting to generalize on the significance of the various reasons due to the limited number of participants. Studies with larger populations are therefore warranted.

The overall number of participants and generated statements involved in the study are in accordance with findings and recommendations in the core literature on the GCM methodology [18]. Similarly, a stress value below the commonly accepted threshold indicated that the sorting phase was sufficient and reliable despite the relatively small sample size.

## Conclusion

In conclusion, this study demonstrates that economic reasons and lack of priority were the primary barriers for pregnant women not seeing a dentist regularly. With the high prevalence of pregnant women not seeing a dentist on a regular basis, identification of these barriers is of public health importance. The results suggested that economic initiatives, giving priority, and dental fear are of the highest importance when introducing changes. Future studies are needed to clarify the promoting factors for seeing a dentist on a regular basis, and to gain information on what could be emphasized further.

## Supplementary Material

Barriers for why pregnant women do not visit a dentist on a regular basis: using group concept mapping methodology

## Data Availability

The data of which the group concept mapping analysis was conducted in the study, consists of the 38 statements that are available in Appendix A as Supplemental Material to this publication.
