# Supplementary material for: Barriers for why pregnant women do not visit a dentist on a regular basis: using group concept mapping methodology
Source: Acta Odontol Scand. 2024 Mar 26;83:40286. doi: 10.1080/00016357.2023.2283198 (PMC11302643; doi:10.1080/00016357.2023.2283198)
Supplement: Barriers for why pregnant women do not visit a dentist on a regular basis: using group concept mapping methodology [file AOS-83-40286-s1.pdf]

Supplementary material has been published as submitted. It has not been copyedited or typeset by Acta Odontologica Scandinavica.

Appendix A Ideas within the 5 clusters, rated for importance **and need for change**

**Cluster**	**Statement**	**Importance median**	**Need for change 'yes' n**
**1 Economic reasons**			
n=10	**1. Primarily because it is expensive**	**3**	**7**
	4. Would like to, but a student budget does not cover it	1	5
	8. It's too expensive	2	7
	10. Besides, I don't think I have the money for it	3	6
	12. After living with only one income for some years, due to illness, is has not been prioritized	1	4
	**14. I don't think I can afford it**	**3**	**7**
	**15. Your never know how much money it would end up being**	**3**	**7**
	27. Because I have had financial challenges for years	2	4
	29. It is too expensive, and I don't have that much money	3	7
	37. It can seem overwhelming because you do not know how expensive it will be	2	7
**2. Priority**			
n=15	2. Because it is not something you "must" do	1	4
	7. I have moved many times*	1	2
	9. I have prioritized wrongly	2	6
	11. The time has not been right for it*	2	3
	13. It has not been re-prioritized again for now	2	5
	**16. I just don't get it done***	**3**	**7**
	17. I don't think so much about it*	2	5
	18. Many times, when you are called in, the time does not fit due to work or other things*	1	1

	20. I have never gotten into the routine of having a regular dental checkup	2	7
	22. I forget*	3	6
	25. I never got the “routine”	2	6
	**26. I forget to make time for that**	**3**	**8**
	31. I do not prioritize it even though it is very important	2,5	5
	32. I keep putting it off	3	6
	34. I don’t prioritize it*	3	6
**3. Lack of time and energy**			
n=8	3. So you do not get called in for checkups or something like that**	2	5
	5. I don’t have the energy for it	2	8
	6. It is difficult to make ends meet in everyday life with work and children	1	3
	19. I cannot be motivated to call in and get a new time and it will be 4 years now. That’s not how I felt before I became a mother	1	5
	28. I have not problems with my teeth and have therefore not been in a hurry	2	3
	30. In a busy everyday life with a job/child of 3 years and a dog, it is just difficult to find time for it all	1	4
	33. It is not so nice to take a little baby with you to the dentist**	2	4
	**35. I forget myself**	**3**	**7**
**4. No problems with teeth**			
n=3	21. I have wanted regular dental checkups but have not dental issues	1	3
n	23. I have not had problems with my teeth	2	2
	24. I have never had or have not had problems with teeth	1	1
**5. Dental fear**			
n=2	36. Previous bad experiences with dentist	1	6
	38. I have a fear of dentists	1	6

Changes during the validation meeting: * statements moved from cluster three to two (n=7), ** statements moved from cluster four to three (n=2).

Statements with a high rating of importance (median $\geq$3) combined with a need for changes ($\geq$ 70% rating ‘yes’) are marked in bold.
